# An Improved Dehydroepiandrosterone-Induced Rat Model of Polycystic Ovary Syndrome (PCOS): Post-pubertal Improve PCOS's Features

**DOI:** 10.3389/fendo.2018.00735

**Published:** 2018-12-04

**Authors:** Eun-Jeong Kim, Minhee Jang, Jong Hee Choi, Kyoung Sun Park, Ik-Hyun Cho

**Affiliations:** ^1^Department of Science in Korean Medicine and Brain Korea 21 Plus Program, Graduate School, Kyung Hee University, Seoul, South Korea; ^2^Department of Convergence Medical Science, College of Korean Medicine, Kyung Hee University, Seoul, South Korea; ^3^Department of Korean Medicine Obstetrics and Gynecology, College of Korean Medicine, Kyung Hee University, Seoul, South Korea; ^4^Institute of Korean Medicine, College of Korean Medicine, Kyung Hee University, Seoul, South Korea

**Keywords:** polycystic ovary syndrome model, dehydroepiandrosterone, post-puberty, rats, improved model

## Abstract

Complete animal models investigating the pathogenesis and treatment of polycystic ovarian syndrome (PCOS) are not completely established. Although dehydroepiandrosterone (DHEA)-induced pre-pubertal rat model for PCOS has been widely used, the model exhibits weaknesses such as decreased ovary weight. Here, we report an innovative DHEA-induced PCOS model that addresses limitations of the pre-pubertal model. The 21-day-old (pre-pubertal) and 42-day-old (post-pubertal) female rats were subcutaneously injected with DHEA (60 mg/kg body weight) daily for up to 20–30 days. The post-pubertal model showed a steady increase in ovary weight and the number of ovarian cysts as well as uterine weight and thickness, which may be key features of PCOS, compared with the pre-pubertal model. Therefore, a post-pubertal PCOS model induced by DHEA may be an improved model to investigate the etiology of PCOS and development of therapeutic interventions.

## Introduction

Polycystic ovary syndrome (PCOS), the most common cause of female subfertility, is estimated to affect 5–10% of women during and beyond their reproductive years (1, 2). Although the etiology of PCOS might be related to genetic and environmental factors such as diet and stress (1–3), the cause of PCOS still remains to be elucidated. PCOS is characterized by chronic menstrual dysfunction (oligo- or anovulation), impaired fertility, hirsutism (excessive body hair growth), acne, obesity, diabetes mellitus, hyper-androgenism, metabolic disturbances (dyslipidemia, hyperinsulinemia, insulin resistance, and type 2 diabetes), enhanced incidence of endometrial hyperplasia and cancer, and polycystic ovaries (1–5). Nonetheless, no appropriate therapeutic strategy for PCOS has yet been established (1–5). Additionally, some animal models failed to provide adequate insight into the pathogenesis of PCOS for evaluation of treatment alternatives (6–11).

Currently, animal models of rats, mice, rhesus monkeys, and ewes are used to study PCOS induced by subcutaneous injection or implantation of androgens, estrogens, anti-progesterone, and letrozole, prenatal exposure to excess androgens, and by exposure to constant light (6–11). Further, spontaneous PCOS-like rodent models (JCR:LA-cp rats), and transgenic (Tg) mouse models (LHr-hIGF-I and bLHb-CTP Tg strains) are used (12–14). Among these, the rat model has been the most widely used for PCOS, due to the small body size, short lifespan, and high reproduction index (6–14), despite limited studies investigating the cardiometabolic aspects and changes in body weight (6, 12–14).

The dehydroepiandrosterone (DHEA)-induced PCOS model of rat has been widely used (6, 7). The blood levels of DHEA, the first androgen to appear during female adolescent years (15), are elevated in 25% of women with PCOS (16). The rodent model of DHEA-induced PCOS was first reported by Roy et al. (15). To induce the PCOS model, pre-pubertal rats (~22 days after birth) are subcutaneously injected daily with DHEA (6 mg/100 g body weight, dissolved in 0.2 mL of sesame oil) for up to 20–27 days (15). After induction, rats displayed several salient features of PCOS including menstrual dysfunction and polycystic ovaries (from 0.45 to 2.2 mm in diameter). Recently, we used this pre-pubertal PCOS model to investigate the protective effect of an Oriental medicine Kyung-Ok-Ko on PCOS (17) and associated uterine abnormality (18). We encountered frequent aplasia of ovary or ovarian cyst, in maintaining high reproducibility in PCOS model induction. Therefore, we sought to improve the previous protocol involving pre-pubertal rats. With the aim to address the aforementioned limitations of the pre-pubertal model, we report an innovative post-pubertal rat model of DHEA-induced PCOS.

## Materials and Metnods

### Animals and Ethical Approval

Sprague Dawley dams, each with 8–10 pups (OrientBio Inc., Seoul, Republic of Korea; Seed mice were originated from Charles River laboratories Japan, Inc., Yokohama-shi. Kanagawa, Japan), were housed at a constant temperature of 23 ± 2°C and humidity of 55–65% with a 12 h light-dark cycle (lights on from 08:00 to 20:00), and provided with commercial chow (21.68% protein, 4.6% fat, 2.45% fibers, 5.97 ash, and 2.5% minerals; Purina Korea Inc., Seongnam-si, South Korea) and water *ad libitum*. All experimental procedures were reviewed and approved by the Institutional Animal Care and Use Committee of Kyung Hee University (KHUASP(SE)-17-072). In this process, proper randomization of laboratory animals and handling of data were performed in a blinded manner in accordance with recent recommendations from an NIH workshop on preclinical models of neurological diseases (19).

### Endpoint Criteria

Since endpoint criteria in DHEA-induced PCOS rat model and DHEA's toxicity to rats was not well known, we established as endpoint criteria for the present study several stems among very general criteria for *in vivo* study (20); The primary endpoint of this study was at least 10% weight loss with <40% food and water intake of sham within 7 days following DHEA treatment. The secondary endpoint was at least 20% weight loss without food or water intake with repeated or prolonged convulsions or ataxia at the end of an experimental period.

### PCOS Induction

Female rats at 21-day-old and 42-day-old were housed separately by age group. Four mice per cage were considered maximum capacity. For pre-pubertal experiment, female rats at 21-day-old (*n* = 56) were allocated 3 or 5 rats for sham and 5 rats for DHEA group. For post-pubertal experiment, female rats at 42-day-old (*n* = 109) were allocated 3 or 5 rats for sham and 4–7 rats for DHEA group. We tried to reduce the number of rat by 3Rs principle (Replacement, Reduction, and Refinement). To reduce effects of animal variation, we selected female rats from littermate or of the same birthday. Rats in the DHEA group were subcutaneously injected daily with DHEA (Millipore, Darmstadt, Germany; 60 mg/kg body weight) dissolved in 0.2 mL sesame oil (Sigma-Aldrich, St. Louis, MO, USA) beginning at 21 and 42 days of age for up to 20–30 days (17). The dose of DHEA was chosen according to a previous study (17). Rats in the sham group were injected with sesame oil without DHEA. Pre-pubertal and post-pubertal experiments were accomplished 6 and 11 times, respectively.

### Body Weight Measurement

Body weight was measured every morning between 8:00 and 9:00 am during the experimental period, before treatment with DHEA or saline.

### Vaginal Smears

To determine the estrous stage, vaginal smear test was performed daily beginning on day 10 (31 days after birth) in the pre-pubertal group and on day 0 (42 days after birth) in the post-pubertal group after DHEA injection as described previously (17). A moistened cotton bud swab was inserted into the vagina. Cells from the vaginal lumen and walls were gently removed and were transferred to a glass slide and air-dried. The air-dried smears were fixed by dipping the smears briefly (two dips) in a Coplin jar containing absolute methanol. The smears were removed from absolute methanol and let air dry. The air-dried smears were stained with diluted Giemsa stain (1:20, vol/vol; YD Diagnostics, Yongin-si, Republic of Korea) for 20 min. For a 1:20 dilution, 2 ml of stock Giemsa was added to 40 ml of buffered water in a Coplin jar. The stained smears were washed by briefly dipping the slide in and out of a Coplin jar of buffered water (one or two dips). The smears were noted to be not decolorized by excessive washing. The stained smears were let air dry in a vertical position. The estrous stage was determined by microscopic analysis of the predominant cell type in vaginal smear as described previously (21). Briefly, proestrus and estrous stages consisted of a predominance of nucleated epithelial cells and anucleated cornified cells, respectively. A metestrus stage consisted of the same proportion among leukocytes, cornified, and nucleated epithelial cells while a diestrus smear primarily consisted of a predominance of leukocytes.

### Histological Examination

Histological examination was carried out as previously described (17, 18). The 24 h after the last DHEA injection, rats were anesthetized with diethyl ether in a large desiccator. The ovaries and uteri were immediately removed through a small ventral midline laparotomy, measured (weight for ovaries and uteri; length for uteri), and fixed at 4°C overnight in fresh 4% paraformaldehyde (Sigma-Aldrich) in 0.1 M phosphate buffer (pH 7.4; Added 3.1 g of NaH_2_PO_4_•H_2_O (Sigma-Aldrich) and 10.9 g of Na_2_HPO_4_ (anhydrous; Sigma-Aldrich) to distilled H_2_O to make a volume of 1 L). Ovaries and middle portions (about 0.5 cm) of the uteri were washed with distilled water for more 6 h at room temperature, dehydrated in graded series of ethanol (Sigma-Aldrich) for 40 min each: 70%, 80%, 90%, 89%, 100% I, and 100% II in distilled water. The ovaries and middle portions of the uteri were cleared in ethanol/xylene 1:1, xylene I, and xylene II for 40 min each, infiltrated with paraffin wax I, II, and III for 40 min each, embedded with melted paraffin wax using stainless steel molds, and placed it on a refrigerated surface. Paraffin blocks were cut into 5-μm-thick longitudinal sections for ovaries and in a transverse plane for uteri using a Leica RM 2155 microtome (Leica Biosystems, Wetzlar, Germany) and air-dried in a vertical position.

The slides were stained with hematoxylin–eosin (H&E) dye. The slides containing paraffin sections in a slide holder (metal) were deparaffinized and rehydrated in xylene I, II, and III, 100% ethanol, 95% ethanol, 80% ethanol, and distilled water for 5 min each. Excess water from slide holder were blotted, dipped in hematoxalin (Harris hematoxylin with glacial acetic acid; s212a; Poly Scientific, Bayshore, NY, USA) for 5 min, rinsed in distilled water, placed top water for 5 min to allow stain to develop, dipped acid ethanol (8–12 times, fast) to destain, rinsed in tap water for 2 min, and rinse distilled water for 2 min. Excess water from slide holder were blotted, dipped in eosin (eosin phloxine alcoholic working solution; s176; Poly Scientific) for 30 s, dipped in 95% ethanol I/II/II, 100% ethanol I/II/III, and Xylene I/II/III for 5 min each. The slides were coverslipped with xylene based permount (Fisher Scientific, Hampton, NH, USA). Images of stained sections were visualized and captured using a DP70 digital light microscope system (Olympus, Tokyo, Japan). The ovarian and uterine morphology was observed microscopically under low/high-power fields in midline sections obtained from ovaries and uteri of each group.

### Statistical Analysis

Results are expressed as mean ± SEM. Mann-Whitney *U*-test (a non-parametric statistical approach) was used to compare the mean body, ovary, and uterus weights between both groups and Fisher's exact test (a non-parametric statistical approach) was used to compare the rates of irregular cycles and polycystic ovary between both groups in SPSS 21.0 package (SPSS Inc, Chicago, USA) for Windows. *P-*values < 0.05 were considered significant.

## Results

### PCOS Model of Pre-pubertal Rats Displays Irregular Phenotypes

First, we examined whether PCOS model from pre-pubertal rats (21 days old) maintains high reproducibility of PCOS features (Table 1 and Figure 1). Since increase in body weight might be one of the major clinical features of PCOS patients (1, 2, 3, 4, 5), we investigated whether DHEA treatment altered the body weight of pre-pubertal rats. A significant increase in body weight was unclear from 21 to 28 days after DHEA injection compared with age-matched sham rats. Only 2 of the 6 trials (33%) displayed significant increase in body weight (Table 1). The mean 72% (18 of the 25 rats) of rats from PCOS group displayed significant irregular estrus cycles and rats from all sham groups displayed regular estrus cycles (Table 1). Since the size of ovaries and uteri from PCOS rats can change (3), we examined them in each group. The ovarian weights in the DHEA group were significantly increased in only 3 of 6 trials (50%) compared with the sham group, but was significantly decreased in 1 trial (16.7%), as shown in Table 1. The increase in the ovarian weight was consistent with the increase in body weight (Table 1). Interestingly, the ovarian appearance and size was regular in the sham group (Figure 1A), but very irregular in the DHEA group (Figure 1B). A few ovaries of the DHEA group were not even fully developed (arrows in the Figure 1B). According to histopathological analysis, the size and number of cysts in the ovaries of DHEA group were generally proportional to their weight and size (Table 1 and Figures 1D–G), compared with the sham rats (Table 1 and Figure 1C). The mean 50% (5 of the 10 rats) of ovaries from DHEA group displayed multi-cystic ovaries and all sham ovaries displayed normal structures (Table 1). The uterine weights and sizes in the DHEA group were significantly increased in only 3 out of 4 trials (75%), compared with the sham group (Table 1). The size of uteri and thickness of uterine wall of DHEA group were generally proportional to their weight and size (Table 1 and Figures 1K–M), compared with the sham rats (Table 1 and Figure 1J).

**Table 1:** Comparison of characters in DHEA-induced PCOS model of pre-pubertal rats.

	**Period of DHEA treatment(day after birth)**	**Mean body weight on end day (g)**		**Ratio (%) of rats with irregular estrus cycles (numbers)**		**Mean ovary weight (mg/each)**		**Ratio of rats with multi–cystic ovaries (numbers)**		**Mean uterus weight (g/cm)**	
		**Sham**	**DHEA**	**Sham**	**DHEA**	**Sham**	**DHEA**	**Sham**	**DHEA**	**Sham**	**DHEA**
Pre–pubertal (60 mg/kg BW, s.c., SD rat)	21–49	197.3 ± 3.4	195.8 ± 5.0	0 (0/3)	80 (4/5)	39.6 ± 1.8	26.5 ± 5.6	0 (0/3)	100^#^ (5/5)	–	–
	21–42	157.7 ± 2.2	153.2 ± 2.3	0 (0/3)	80 (4/5)	30.0 ± 1.2	23.3 ± 4.7	0 (0/3)	0 (0/5)	–	–
	21–42	172.5 ± 3.8	170.8 ± 2.6	– (N/5)	– (N/5)	34.2 ± 5.2	17.9 ± 1.7^#^	– (N/5)	– (N/5)	47.9 ± 10.9	61.7 ± 4.3
	21–42	141.4 ± 2.4	166.1 ± 2.3^#^	0 (0/5)	80^#^ (4/5)	33.4 ± 1.6	51.4 ± 2.3^#^	– (N/5)	– (N/5)	38.1 ± 2.4	64.0 ± 7.2^#^
	21–46	148.3 ± 2.0	168.0 ± 1.8^#^	0 (0/5)	60 (3/5)	35.9 ± 2.6	52.9 ± 2.7^#^	– (N/5)	– (N/5)	41.8 ± 1.2	81.3 ± 10.6^#^
	21–46	145.4 ± 4.2	157.4 ± 4.5	0 (0/5)	60 (3/5)	35.3 ± 2.3	52.5 ± 2.8^#^	– (N/5)	– (N/5)	38.9 ± 1.9	59.5 ± 7.5^#^
Mean	–	–	–	0 (0/21)	72^##^ (18/25)	–	–	0 (0/6)	50 (5/10)	–	–

# p < 0.05 and

## *p < 0.01 vs. Sham group. The “-” and “N”, not be identified*.

**Figure 1:**
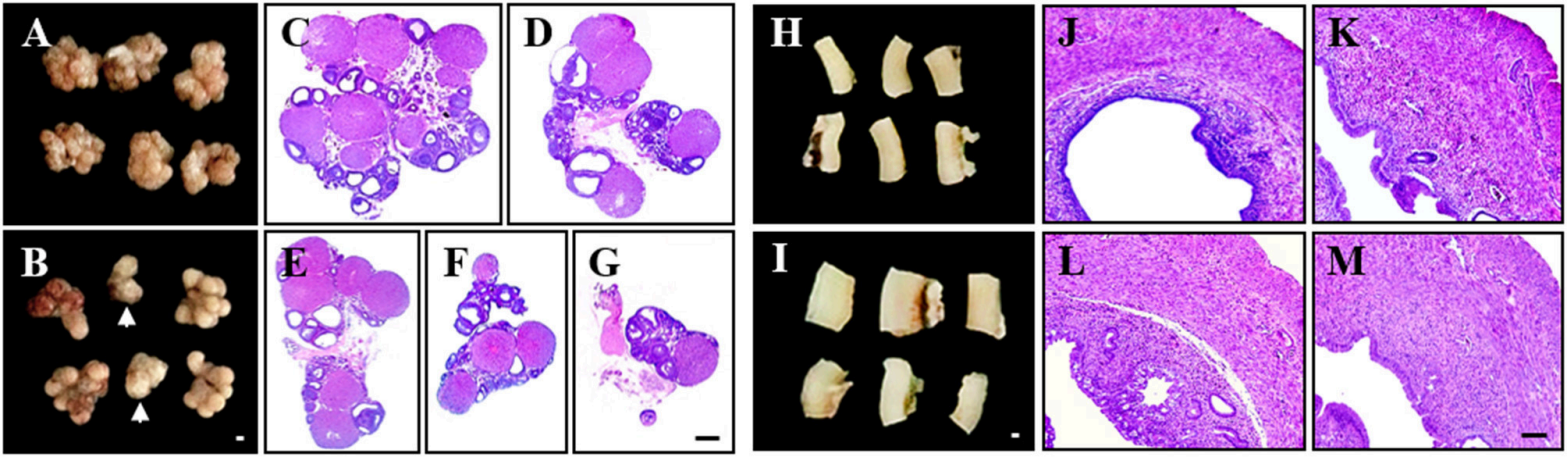
Irregular phenotypes in pre-pubertal PCOS model. The pre-pubertal (21-day-old) female rats were subcutaneously injected daily with DHEA for up to 21 days. Rats in the sham group were injected with sesame oil without DHEA. **(A,B)** Ovarian appearance from sham **(A)** and DHEA groups **(B). (C–G)** Paraffin sections from sham **(C)** and DHEA groups **(D–G)** were stained H&E dye. **(H,I)** Uterine appearance from sham **(H)** and DHEA groups **(I)**. **(S–D)** Paraffin sections from sham **(J)** and DHEA groups **(K–M)** were stained H&E dye. Scale bars in **(B,G,I,M)** = 500 μm.

### PCOS Model of Post-pubertal Rats Displays Improved Phenotypes

**Figure 2:**
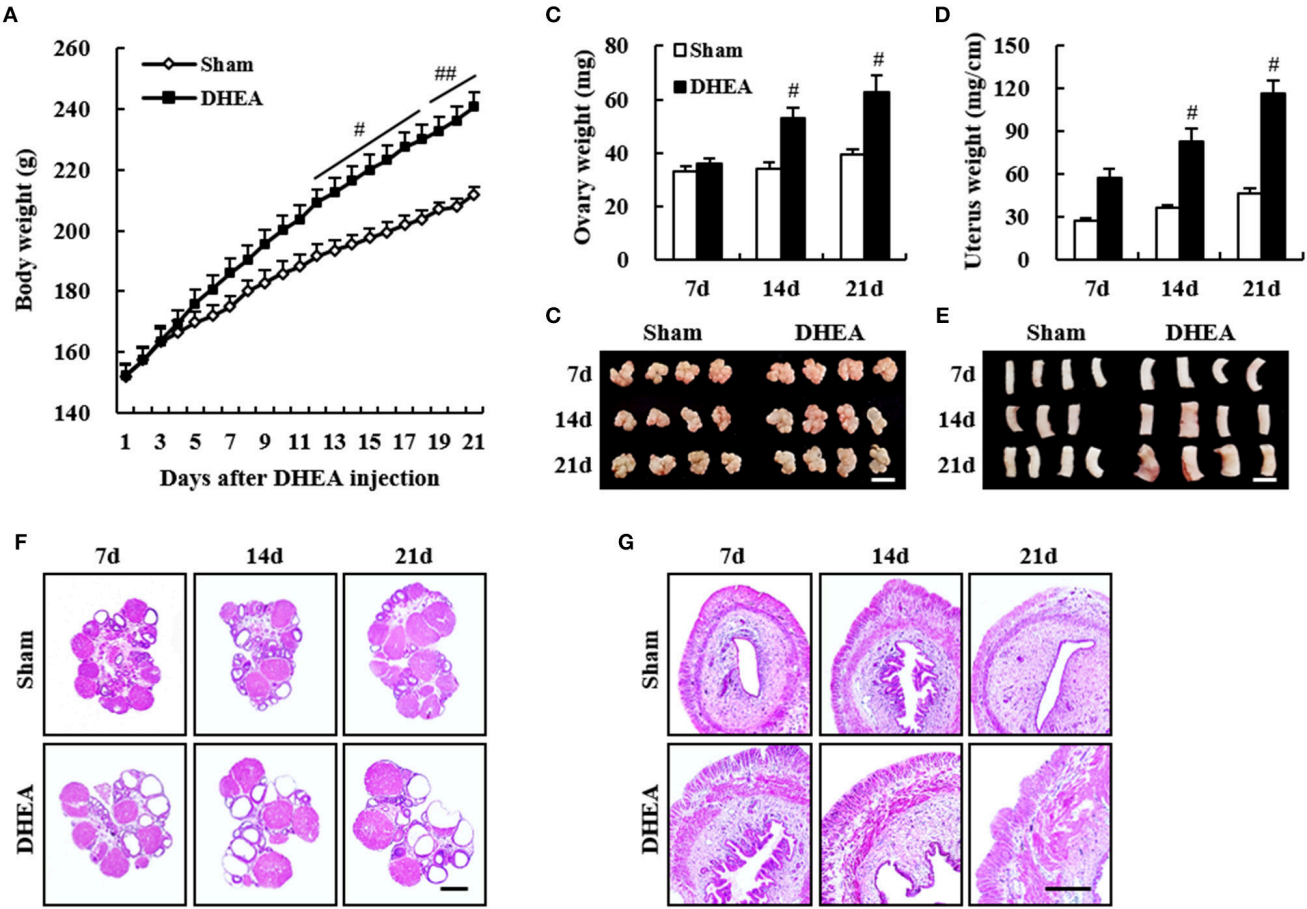
Improved phenotypes in post-pubertal PCOS model. The post-pubertal (42-day-old) female rats were subcutaneously injected daily with DHEA for up to 20 days. Rats in the sham group were injected with sesame oil without DHEA. **(A)** Change in body weight after DHEA injection. **(B,D)** Change in ovary weight **(B)** and uterus weight **(D)** after DHEA injection. **(C,E)** Change in appearance of ovaries **(C)** and uteri **(E)** after DHEA injection. **(F,G)** Paraffin sections from sham (upper panel) and DHEA groups (lower panel) were stained H&E dye. Scale bars in **(C,E,F,G)** = 500 μm. Mann Whitney U test; ^#^*p* < 0.05 and ^##^*p* < 0.01 vs. Sham group.

To develop a DHEA model with increased reproducibility of PCOS features, compared with pre-pubertal rats, we developed a PCOS model by injecting DHEA into post-pubertal rats (42 days old; Figure 2). The body weight from the DHEA group was significantly increased 12–21 days after DHEA injection, compared with that of the sham group (Figure 2A). The ovarian weights from the DHEA group were significantly increased at 14 and 21 days after DHEA injection, compared with the sham group (Figure 2B). Consistent with these results, the ovarian size was also increased (Figure 2C). Histopathological analysis confirmed ovarian cysts starting 7 days after DHEA injection, with gradually increasing size and number until day 21 (Figure 2F). The uterine weights from the DHEA group were significantly increased since day 7 after DHEA injection and gradually increased until day 21, compared with the sham group (Figure 2D). Consistent with these results, the uterine size was also increased (Figure 2E). Histopathological analysis revealed a gradual increase in the thickness of uterine wall since day 7 after DHEA injection (Figure 2G).

Therefore, we evaluated whether the DHEA model of post-pubertal rats improved repeatability and reproducibility of the disease (Table 2). The body weight of rats in the DHEA group displayed significant changes only in 5 out of 11 trials (45.5%) 20–30 days after DHEA injection, compared with the sham group (Table 2). The mean 87.5% (28 of the 32 rats) of rats from PCOS group displayed significant irregular estrus cycles and all the rats in the sham group displayed regular estrus cycles (Table 2). The ovarian weight in the DHEA group significantly increased in 6 out of 8 trials (90.9%) compared with the sham group. In the DHEA group, the mean 85.9% (49 of the 57 rats) of ovaries showed significant multi-cystic ovaries compared with normal structures in all sham ovaries (Table 2). The uterine weight in the DHEA group of rats significantly increased in 10 out of 11 trials (90.9%), compared with the rats in the sham group (Table 2).

**Table 2:** Comparison of characters in DHEA-induced PCOS model of post-pubertal rats.

	**Period of DHEA treatment(day after birth)**	**Mean body weight on end day (g)**		**Ratio (%) of rats with irregular estrus cycles (numbers)**		**Mean ovary weight (mg/each)**		**Ratio of rats with multi–cystic ovaries (numbers)**		**Mean uterus weight (g/cm)**	
		**Sham**	**DHEA**	**Sham**	**DHEA**	**Sham**	**DHEA**	**Sham**	**DHEA**	**Sham**	**DHEA**
Pubertal (60 mg/kg BW, s.c., SD rat)	42–63	247.0 ± 9.7	243.4 ± 5.7	0 (0/4)	100^#^ (4/4)	39.7 ± 1.9	57.0 ± 1.5^#^	0 (0/4)	75 (3/4)	46.6 ± 2.7	116.8 ± 8.6^#^
	42–63	245.5 ± 7.5	249.2 ± 4.9	0 (0/4)	100^##^ (5/5)	37.6 ± 1.1	67.8 ± 6.6^#^	0 (0/4)	80^#^ (4/5)	47.3 ± 5.6	103.4 ± 15.4^#^
	42–62	266.2 ± 9.4	264.4 ± 6.9	0 (0/5)	100^##^ (6/6)	44.4 ± 5.0	62.5 ± 4.6^#^	0 (0/5)	100^##^ (6/6)	44.9 ± 2.2	105.2 ± 1.7^##^
	42–70	267.5 ± 10	254.8 ± 4.1	0 (0/4)	100^#^ (4/4)	37.5 ± 1.5	77.6 ± 9.1^#^	0 (0/4)	75 (3/4)	51.5 ± 4.3	127.8 ± 12^#^
	42–72	282.3 ± 11.6	242.4 ± 14.0	0 (0/5)	70^#^ (5/7)	49.5 ± 3.2	47.7 ± 6.4	0 (0/5)	70^#^ (5/7)	71.2 ± 12.7	91.7 ± 7.4
	42–72	251.6 ± 7.9	250.0 ± 5.9	0 (0/5)	66.7 (4/6)	47.5 ± 2.1	55.1 ± 2.6^#^	0 (0/5)	100^##^ (6/6)	71.6 ± 2.5	121.3 ± 4.3^#^
	42–62	219.3 ± 2.8	243.5 ± 4.0^#^	– (N/5)	– (N/5)	36.5 ± 2.0	63.4 ± 3.2^##^	0 (0/5)	80^#^ (4/5)	54.0 ± 1.4	97.4 ± 3.0^#^
	42–62	245.9 ± 2.2	267.4 ± 4.6^#^	– (N/5)	– (N/5)	49.1 ± 3.2	67.9 ± 6.1	0 (0/5)	100^##^ (5/5)	63.3 ± 5.4	105.2 ± 7.0^#^
	42–62	241.4 ± 5.6	262.9 ± 5.0^#^	– (N/5)	– (N/5)	39.7 ± 1.9	56.6 ± 1.5	0 (0/5)	100^##^ (5/5)	67.3 ± 15.4	129.2 ± 4.0^#^
	42–62	235.4 ± 3.1	257.9 ± 5.8^#^	– (N/5)	– (N/5)	46.7 ± 1.2	62.7 ± 4.3^##^	0 (0/5)	80^#^ (4/5)	64.1 ± 6.1	109.5 ± 7.5^#^
	42–62	223.7 ± 4.5	245.5 ± 0.9^#^	– (N/5)	– (N/5)	41.1 ± 4.1	67.0 ± 9.3^#^	0 (0/5)	80^#^ (4/5)	60.6 ± 2.7	101.3 ± 5.3^##^
Mean	–	–	–	0 (0/27)	87.5^##^ (28/32)	–	–	0 (0/52)	85.9^##^ (49/57)	–	–

# p < 0.05 and

## *p < 0.01 vs. Sham group. The “-” and “N”, not be identified*.

## Discussion

Various PCOS animal models, including rodents (mouse and rat), sheep, and primates, have been developed over several decades (6–12). Although the histopathological and metabolic features of PCOS rodent models do not always coincide with those in humans, the rodent models are still useful in studying the pathogenesis of and therapeutic interventions for PCOS (6–12). Therefore, it is worth improving the current PCOS models by developing breakthrough models. We detected low reproducibility and irregular induction ratio in pre-pubertal rat models with DHEA (Table 1 and Figure 1) while screening new materials for PCOS. Thus, we improved the limitations of the pre-pubertal model by developing post-pubertal rat models induced with DHEA (Table 2 and Figure 2). The post-pubertal rat model displayed increased ratio of rats with irregular estrus cycles (66.7–100%) and multi-cystic ovaries (75–100%) as well as increased ovarian weight and uterine weights/thickness compared to the pre-pubertal model (Table 2 and Figure 2). Therefore, our post-pubertal PCOS model induced by DHEA may be an improved model to investigate the etiology of PCOS and development of therapeutic interventions.

In the present study, the post-pubertal rat model reproducibly induced the formation of follicular cysts in ovaries which appeared at a higher frequency when the treatment of DHEA was started at an adolescence age of 42 days compared to pre-pubertal rat model (Table 1 and Figure 1). Such higher frequency continued for 20–30 consecutive days (until 42–72 days-of-age; Table 2 and Figure 2). Puberty (estrous cycle) of rat and human starts at 35 to 40 days of age (22) and 12–14 years, respectively, after birth. Our findings suggest that the post-pubertal rat model may increase reproducibility of the DHEA-induced PCOS model. Such effect might be closely related to PCOS in adolescents and early onset of PCOS in young adults (23, 24, 25). However, further research is needed to identify endocrinological, histopathological, and molecular biological mechanisms of DHEA in post-pubertal rat model as a rodent model for human PCOS.

The PCOS rodent model using postnatal (pre-pubertal) DHEA treatment (11, 15) was developed by increasing the DHEA levels of PCOS patients (11, 15, 26). Treatment of pre-pubertal rats and mice with DHEA for 20 consecutive days induces major features in women with PCOS, such as irregular menstruation, abnormal maturation of follicles, anovulation, and amenorrhea (11, 15, 26–28). However, in the present study, the pre-pubertal rat model with DHEA exhibited a mixture of rats with irregular estrus cycles mean 72% and multi-cystic ovaries mean 50% displaying altered ovarian and uterine weights (Table 1 and Figure 1). Some ovaries were not developed normally and regressed (Figure 1B). These results may be explained by two conflicting reports. In one study, luteinizing hormone (LH) levels were elevated while follicle-stimulating hormone (FSH) levels did not change (11, 27), whereas in other studies, LH and FSH levels were both decreased (11) or unchanged (29). Limited data are available to determine metabolic disturbances associated with PCOS following DHEA treatment. In the present study, these adverse effects in pre-pubertal rats treated with DHEA were obviously improved in the post-pubertal rats. The ratio of rats with irregular estrus cycles and multi-cystic ovaries were mean 87.5% and mean 85.9%, respectively, and ovarian and uterine weights increased evenly (Table 2 and Figure 2). The results suggest that pre-pubertal exposure to androgens disturb sexual maturity whereas exposure during or after puberty induces more stable PCOS. Collectively, the post-puberty model represents a useful tool to study PCOS during or after puberty.

Prenatal exposure to androgens induces vaginal fusion (30). Therefore, researchers experimented with various doses of androgens to minimize this effect. However, this approach was a significant limitation of this model for evaluation of fertility, which is a key feature of patients diagnosed with PCOS (30). Furthermore, a few neonatally androgenized rats showed elevated androgen and LH levels; however, the expression level was not consistent, and a few models displayed normal serum levels of LH, FSH, T, and E2 (31). Meanwhile, postnatal exposure to androgens decreased ovarian weight, in contrast to the enhanced size of ovaries in PCOS patients (11, 31). The results may support features including low ratio in irregular estrous cycle, low ratio in multi-cystic ovaries, and irregular weight changes in ovary and uterus in the pre-puberty model of the present study (Table 1 and Figure 1) disputing their relevance as PCOS models. However, the post-puberty model in the present study displayed a stable increase in ovarian (and uterine) weight and size, and increased homogeneity of ovarian cyst formation and uterine abnormality, which are key features of PCOS (Table 2 and Figure 2). Collectively, our post-puberty PCOS model improved the limitations of previous PCOS models.

Many patients with PCOS have features of metabolic syndrome, including insulin resistance, obesity, and dyslipidemia (1–5). However, its pathogenesis is still poorly understood. PCOS is hypothesized to result from the interaction of genetic and environmental factors, manifesting initially in the presence of mature gonadotropin levels at puberty (32). After prenatal exposure to high levels of androgen/testosterone, adolescent and adult sheep and rhesus monkeys display various features of PCOS. However, the exorbitant price of medium-sized animals prevents studies investigating the pathogenesis of PCOS using them (32–34). Environmental factors (over-nutrition and industrial products, particularly Bisphenol A) may increase the risk of PCOS clinical phenotype in susceptible women. The rodent model has been the most widely used for PCOS, with advantages including small body size, short lifespan, and high reproductive index (6–11). Therefore, the rat PCOS model by DHEA facilitates the study of PCOS.

## Conclusions

Overall, rodent models facilitate our understanding of the pathogenesis of PCOS and development of potential therapeutics for PCOS. However, each model has its own merits and demerits, and individually cannot meet all the conditions associated with the pathological status of PCOS. In the present study, post-pubertal exposure to excessive DHEA improved reproducibility, PCOS induction ratio, ovarian cyst formation, and uterine malformation. Therefore, our PCOS model might contribute to the research and development of PCOS.

## Author Contributions

E-JK performed model induction and histopathological analysis, and prepared the figures. MJ and JHC assisted with H&E staining and animal maintenance. KSP analyzed and commented the results. I-HC conceived all experiments, analyzed the results, and wrote the manuscript. All authors have read and approved the final manuscript.

### Conflict of Interest Statement

The authors declare that the research was conducted in the absence of any commercial or financial relationships that could be construed as a potential conflict of interest.
